# Transformational Nurse Leaders and Nurse Well-Being: Examining Mediating Influences of Organizational Justice and Quality of Work Life Mixed Methods Study

**DOI:** 10.1155/jonm/4577350

**Published:** 2025-04-01

**Authors:** Abeer Nuwayfi Alruwaili

**Affiliations:** Department of Nursing Administration and Education, College of Nursing, Jouf University, Sakaka 72388, Saudi Arabia

**Keywords:** mixed method, nurse well-being, organizational justice, quality of work life, transformational leadership

## Abstract

**Background:** Transformational leadership is recognized as a crucial factor influencing nurses' work experiences and well-being. However, the mechanisms through which transformational leadership affects nurse well-being, especially concerning organizational justice and quality of work life, are not fully understood.

**Aim:** This study investigates the direct and indirect effects of transformational leadership on nurse well-being, focusing on the mediating roles of organizational justice and quality of work life.

**Methods:** Using a sequential explanatory mixed methods design, quantitative data were collected from 580 nurses across five hospitals in Saudi Arabia with validated tools: the Global Transformational Leadership Scale, Organizational Justice Scale, Quality of Nursing Work–Life Scale, Emotional Exhaustion Subscale of the Maslach Burnout Inventory, Job Satisfaction Scale, and Perceived Stress Scale. Qualitative data were obtained from 25 focus group discussions using a validated semistructured discussion guide. Quantitative analyses included hierarchical multiple regression and mediation analyses, while qualitative data wereanalyzed thematically.

**Results:** Transformational leadership significantly reduced emotional exhaustion (*β* = −0.48, *p* < 0.001), increased job satisfaction (*β* = 0.53, *p* < 0.001), and lowered perceived stress (*β* = −0.46, *p* < 0.001). Mediation analyses showed significant indirect effects through organizational justice and quality of work life (indirect effects: −0.34 to 0.38, *p* < 0.001). Qualitative findings highlighted the importance of inspirational motivation, individualized consideration, intellectual stimulation, and idealized influence.

**Conclusions:** Transformational leadership directly and indirectly enhances nurses' well-being through improved organizational justice and quality of work life. These findings emphasize the need for healthcare organizations to foster transformational leadership and promote fair, supportive work environments to enhance nurse well-being.

## 1. Introduction

In the dynamic landscape of healthcare, nursing plays a pivotal role in shaping patient outcomes and overall health service effectiveness [1–4]. As frontline caregivers, nurses work in demanding environments characterized by long hours, emotional exhaustion, and physical demands, making their well-being a paramount concern [5, 6]. In this context, leadership, particularly transformational leadership, has emerged as a critical factor influencing nurses' work experiences and well-being [3, 7]. Transformational nurse leaders, known for their ability to inspire, motivate, and cultivate a supportive work environment, have the potential to significantly impact employee satisfaction, commitment, and productivity [8–12]. This leadership style transcends traditional behavioral approaches by emphasizing vision, communication, and professional capacity-building [13]. Transformational leadership has been linked to numerous positive outcomes, including increased job satisfaction, reduced turnover, and improved patient care [13, 14]. However, the specific mechanisms through which transformational leadership influences nurse well-being, particularly concerning organizational factors such as justice and quality of work life (QWL), remain underexplored [3, 15, 16].

Nurse well-being is a multidimensional construct encompassing physical, emotional, and mental health aspects [12–15]. It is not only crucial for nurses' personal health but also has significant implications for the quality of patient care they deliver [17, 18]. A growing body of research has investigated various factors contributing to nurse well-being, highlighting the importance of work environment, organizational support, and job characteristics [19–22]. These factors have been shown to significantly impact nurses' job satisfaction, stress levels, and burnout, underscoring the need for a deeper understanding of the determinants of nurse well-being [23–27]. Organizational justice, which refers to the perceived fairness of behaviors within an organization, has emerged as a key factor influencing employee satisfaction and morale [28, 29]. In healthcare settings, where ethics and fairness are highly valued, perceptions of justice can significantly impact staff engagement, commitment, and performance [29, 30]. Similarly, QWL in nursing, encompassing aspects such as job security, work–life balance, and opportunities for professional growth, plays a vital role in shaping nurses' work experiences and well-being [31–35].

Existing research suggests that the effects of leadership and work environment on well-being can vary across different dimensions within broader constructs. For example, specific dimensions of transformational leadership, such as inspirational motivation or individualized consideration, may have different impacts on job satisfaction or emotional exhaustion. Similarly, aspects of organizational justice, including distributive, procedural, and interactional justice, have been shown to uniquely influence employee perceptions of fairness and well-being outcomes. Therefore, separating and analyzing these dimensions individually provide a more granular understanding of how distinct factors contribute to nurse well-being and allow for more targeted interventions to improve workplace outcomes. This approach is supported by studies such as [36–40], which highlight the importance of dimensional analysis in leadership and well-being research.

The intersection of transformational leadership and nurse well-being has garnered considerable attention in nursing research, emphasizing the critical role of effective leadership in fostering positive work conditions and promoting staff well-being [41–44]. Transformational leadership, characterized by leaders who inspire, challenge, and support their followers, has been associated with various positive outcomes in healthcare settings, including increased job satisfaction, reduced burnout, and enhanced team cohesion [45, 46]. Several studies have reported that nurses working under transformational leaders experience higher levels of job satisfaction and well-being, attributing this to this leadership style's supportive and empowering nature [47–50]. Furthermore, organizational justice and QWL have been identified as essential factors influencing nurse satisfaction and retention [51]. Organizational justice, which refers to the perceived fairness of organizational processes and decision-making, has been positively associated with employee well-being and job satisfaction in healthcare settings [52–54]. Likewise, QWL, encompassing elements such as work environment, job demands, and autonomy, have positively correlated with nurses' well-being and job satisfaction [55, 56].

Despite the advancements in understanding the relationships between transformational leadership, organizational justice, QWL, and nurse well-being, a significant gap in the literature persists [57]. While transformational leadership is recognized as enhancing work conditions by promoting perceptions of justice and thereby fostering nurses' positive well-being, the nuanced mechanisms through which these effects operate remain unexamined [38, 58]. Specifically, there is a paucity of comprehensive studies investigating the mediating roles of organizational justice and QWL in the relationship between transformational leadership and nurse well-being [59, 60].

Moreover, much of the existing research is based on cross-sectional designs and quantitative measures, which may not fully capture nursing professionals' dynamic and complex experiences [23, 27, 38, 39, 60]. There is a significant shortage of mixed-method studies incorporating qualitative insights to provide a broader understanding of how nurses perceive and experience the effects of leadership practices, organizational justice, and work–life balance on their well-being [43, 61–64]. This research gap highlights the need for a more holistic approach to exploring these relationships, which requires studies that quantify these interactions while elucidating nurses' subjective experiences and perceptions within diverse healthcare settings [65–68]. Although previous research has established links between transformational leadership, perceptions of organizational fairness, quality of nursing work environments, and nurse well-being outcomes, few studies have simultaneously tested these relationships or explored intermediary effects of justice perceptions and QWL [31–33, 38, 39, 48]. Understanding whether and how transformational leaders shape nurse well-being through these mediating mechanisms is critical, given the central role of nurse managers in ensuring a healthy, engaged, and productive nursing workforce [69–71].

This mixed methods' study addresses these research gaps by investigating organizational justice's and QWL's mediating influences on the relationship between transformational leadership and nurse well-being. By collecting survey data from hospitals to test theorized structural models and qualitative insights directly from nurses through focus groups, this study will elucidate whether the positive effects of transformational leadership style on nurse-reported well-being are transmitted through enhanced perceptions of fairness and enriched QWL. The significance of this study lies in its potential to provide valuable guidance for leadership development initiatives, workforce policies, and strategies to improve nurse job satisfaction [72, 73]. By holistically examining the dynamics shaping the impact of nurse leaders through nurses' lived experiences while employing rigorous quantitative analyses, this research will contribute to a more comprehensive understanding of the complex interplay between leadership, organizational factors, and nurse well-being.

In conclusion, the study adopts a mixed-method approach that integrates a framework for determining theory testing with inductive and comparative case analysis to investigate the complex interaction between transformational leadership, organizational justice, QWL, and well-being of nurses. Through an in-depth examination of the mediator role of organizational justice and QWL, this research contributes to the development of evidence-based interventions and policies to improve the well-being of nurses and improve the quality of medical care. The findings will have important implications for healthcare organizations' human resource management, leadership development, and evidence-based decision-making. By fostering an organizational culture that prioritizes the health and well-being of the nursing workforce, healthcare institutions can optimize their organizational effectiveness and ultimately improve patient outcomes. The multifaceted nature of this study and its potential to inform practical strategies make it a valuable contribution to the field of nursing research and healthcare management.

## 2. Materials and Methods

### 2.1. Theoretical Framework

**Figure 1:**
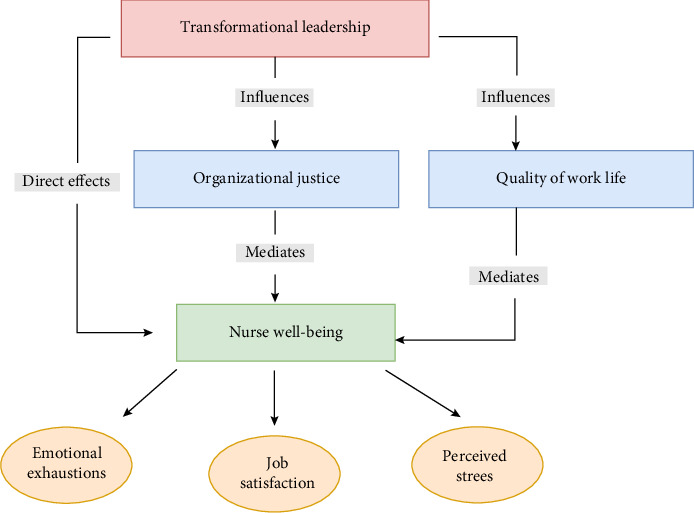
A conceptual model of the direct and indirect effects of transformational leadership on nurse well-being mediated by organizational justice and quality of work life.

This study integrates transformational leadership theory (Bass, 1985) [69, 74] and social exchange theory [75–77] to investigate the influence of transformational nursing leadership on nurses' well-being, with organizational justice and QWL as mediating factors. The proposed theoretical model informed the selection of validated measurement instruments, ensuring alignment with the study's objectives and capturing key constructs such as transformational leadership, organizational justice, QWL, and nurse well-being. The conceptual model (Figure 1) presents transformational leadership as the independent variable, hypothesized to impact nurse well-being directly, and the dependent variable, assessed through measures of emotional exhaustion, job satisfaction, and perceived stress.

The theory of social exchange [77] provides a framework for understanding the relationship between transformational leadership and nurse well-being. This theory posits that reciprocal exchanges between leaders and employees promote positive outcomes. Transformational leadership behaviors foster these exchanges by promoting organizational justice and enhancing the QWL, leading to improved well-being outcomes for nurses [75, 76]. Nurse well-being is defined as a multidimensional construct that includes physical, emotional, and psychological health. It is measured through indicators such as emotional exhaustion, job satisfaction, and perceived stress, which are critical for assessing the overall well-being of nursing staff. Burnout, perceived stress, and job satisfaction were chosen based on their prevalence in literature as key components of well-being, especially in high-stress environments like nursing. In addition, there is substantial evidence suggesting an interaction between organizational fairness and the quality of the work environment, both of which jointly contribute to nurse well-being. Research by Adamovic [34] has demonstrated that organizational fairness positively moderates the effects of a high-quality work environment, reducing burnout and improving job satisfaction [77].

The model also proposes that transformational leadership indirectly influences nurse well-being through organizational justice and QWL mediating variables. Transformational leadership theory suggests that leaders who exhibit inspirational motivation, individualized consideration, idealized influence, and intellectual stimulation can elevate followers' motivation, morale, and performance [74, 78]. In nursing, transformational leaders are expected to create a supportive work environment, promote fairness, and enhance nurses' work experience [78–80]. Social exchange theory provides a complementary perspective. It proposes that nurses who perceive their leaders as transformative are more likely to experience a sense of justice and improved work–life balance [81–83], motivating them to reciprocate through increased commitment, job satisfaction, and well-being [84–86]. These theories provide a comprehensive framework for understanding the complex relationship between transformational leadership, organizational justice, QWL, and nurses' well-being, acknowledging both direct and indirect pathways [78–80].

This study uses mixed methods to empirically test this theoretical model, combining a survey of nurses from several hospitals with comparative focus groups. This approach allows rigorous testing of hypothesized relationships and provides insights into the subjective experiences of nurses. The quantitative component examines the direct and indirect effects of transformational leadership on the well-being of nurses. At the same time, the qualitative dimension provides a deeper understanding of the effect of leadership practices on nurses' well-being through organizational justice and QWL. This study will help develop targeted interventions and programs that promote a positive working environment for nurses by studying the mechanisms by which transformational leadership influences the well-being of nurses. The findings can be used in leadership development programs, staff management strategies, and organizational structures that give priority to the well-being of nursing staff, ultimately improving patient care and healthcare delivery.

### 2.2. Research Hypotheses

  H1: Transformational nurse leaders will be positively associated with improved nurse well-being, as evidenced by reduced emotional exhaustion, increased job satisfaction, and lower perceived stress.
  H2: Organizational justice will significantly mediate the relationship between transformational leadership and nurse well-being. Higher perceptions of fairness fostered by transformational leadership will be associated with improved well-being outcomes among nurses.
  H3: QWL will significantly mediate the relationship between transformational nurse leaders and nurse well-being, with higher QWL under transformational leadership being associated with better well-being outcomes among nurses.
  H4: The combined mediating effects of organizational justice and QWL will significantly contribute to the overall impact of transformational leadership on nurse well-being, offering a comprehensive explanation of how leadership influences nursing staff outcomes.

### 2.3. Design

This study employs an explanatory sequential mixed methods design to investigate the relationships between transformational leadership, organizational justice, QWL, and nurse well-being. The research framework consists of the following two phases:

- Phase 1 (quantitative): A cross-sectional survey design will be used to collect data from a large, representative sample of nurses across multiple hospitals in Saudi Arabia. Validated instruments will measure the key study variables, including transformational leadership, organizational justice, QWL, and nurse well-being indicators (e.g., emotional exhaustion, job satisfaction, and perceived stress). Appropriate statistical methods, such as structural equation modeling (SEM) and mediation analyses, will test the direct and indirect hypothesized relationships in the proposed theoretical model.
- Phase 2 (qualitative): A subset of participants will be purposively selected for focus group discussions following the quantitative phase. These discussions will utilize a semistructured guide to explore nurses' experiences and perceptions regarding transformational leadership, organizational justice, QWL, and their well-being. Thematic analysis will be employed to identify key patterns and themes that provide context and explain the quantitative findings.
- Integration: In the interpretive phase, the quantitative and qualitative findings will be integrated using a joint display approach. This will involve creating matrices or diagrams that juxtapose the quantitative results with the relevant qualitative themes, facilitating a comprehensive understanding of the complex interplay between transformational leadership, organizational justice, QWL, and nurse well-being.

The explanatory sequential design was chosen for its ability to leverage the strengths of both quantitative and qualitative methods, providing a robust and comprehensive understanding of the research problem. The quantitative phase will establish the relationships among the variables. In contrast, the qualitative phase will offer deeper insights into nurses' lived experiences and perceptions, contributing to a more nuanced interpretation of the quantitative findings.

### 2.4. Settings

The study was conducted in five hospitals in the Jouf region of Saudi Arabia. Hospitals were selected based on size, diversity of patient populations, and willingness to participate in the research. These healthcare facilities provide a wide range of medical services, including internal medicine, pediatrics, surgery, obstetrics and gynecology, cardiology, neuroscience, and emergency medicine. They also provide advanced diagnostic facilities, such as imaging systems, laboratory labs, and pathology departments, which are embedded within the hospitals. The multihospital nature of the study allowed for a more representative sample of nurses working in various healthcare settings, increasing the generalizability of the findings. Furthermore, this approach provided an opportunity to compare nurses' experiences and perceptions across diverse organizational settings. The diversity of healthcare systems and nursing experiences represented by the five participating hospitals provided an ideal setting for this study, aiming to contribute to a better understanding of the factors affecting nurses' well-being in the Saudi Arabian healthcare context.

### 2.5. Participants and Sample

The study employed a two-stage sampling approach for participant selection in the mixed methods research design. In the quantitative phase, a stratified random sampling method was used to ensure adequate representation of nurses from each of the five participating hospitals in the Jouf region of Saudi Arabia. This method allowed for accurate identification of the distribution of nurses within healthcare facilities. Within each hospital (stratum), a simple random sampling method was applied to select individual nurses, ensuring equal chances of selection, reduced selection biases, and improved representativeness. The sample size for each hospital was proportionally stratified, resulting in a final sample of 580 nurses, with an average of 116 participants per center. To avoid center effects, random sampling within each center was employed, and multilevel modeling was used in the data analysis to account for potential differences across centers. Registered nurses with at least 1 year of work experience and direct involvement in patient care were eligible to participate, while nurses in nonclinical roles or with less than 1 year of experience were excluded.

The sample size for the quantitative component was determined through power analysis using G ∗ Power software, considering a desired statistical power of 0.90, medium effect size (*f*^2^ = 0.15), and a significance level of *α* = 0.05. To further enhance statistical robustness and account for potential nonresponse, the target sample size was increased to 580 participants, ensuring sufficient power to detect significant effects across the measured constructs. Although the main constructs of the study were transformational leadership, organizational fairness, quality of work environment, and nurse well-being, these constructs were operationalized into 10 measurable variables for the statistical analysis. For instance, nurse well-being was measured using emotional exhaustion, job satisfaction, and perceived stress, while organizational fairness was assessed through distributive, procedural, and interactional justice. This expansion into 10 variables informed the sample size calculation, ensuring robust statistical power. Based on the power analysis, a minimum sample size of 400 nurses was considered necessary. However, to account for potential nonresponse and incomplete data, the target was increased to 480 nurses, and the final sample of 580 nurses provided a robust basis for statistical analysis, allowing for greater generalizability of the findings.

In the qualitative phase, a purposive sampling method was used to select a diverse representation of 25 nurses from the quantitative participants, ensuring diversity in age, gender, work experience, and clinical setting. Participants for the qualitative phase were purposively selected from the quantitative cohort to ensure diversity in demographic characteristics, clinical experience, and work settings, providing a rich, representative sample for thematic analysis. This sample size was chosen to allow in-depth exploration of multiple perspectives during focus group discussions while maintaining manageable group sizes.

### 2.6. Eligibility Criteria

The study employed specific eligibility criteria to select an appropriate sample of participants. The inclusion criteria were as follows: (1) registered nurses currently employed in one of the five participating hospitals in the Jouf region of Saudi Arabia; (2) nurses with at least 1 year of work experience in their current hospital; (3) nurses directly involved in patient care in clinical departments such as internal medicine, surgery, pediatrics, obstetrics and gynecology, and emergency care; and (4) nurses willing to participate in the study and provide informed consent. Exclusion criteria included the following: (1) nurses in nonclinical roles, such as administrative positions or quality assurance departments, and (2) nurses on extended leave, such as maternity leave or long-term sick leave, during the data collection period.

### 2.7. Data Collection Tools

This mixed methods' study utilized both quantitative and qualitative tools to comprehensively explore transformational leadership, organizational justice, QWL, and nurse well-being.

#### 2.7.1. Quantitative Data Collection Tools

The quantitative component utilized a comprehensive survey battery composed of the following validated self-report instruments. To ensure clarity and facilitate understanding of the correlations, the scoring methods for each scale have been detailed as follows:

A. Global Transformational Leadership (GTL) Scale [87]: This seven-item scale assesses staff perceptions of transformational leadership behaviors exhibited by their supervisors across six dimensions: vision, staff development, supportive leadership, empowerment, innovative thinking, and charisma. Each item is rated on a five-point Likert scale, with higher scores indicating stronger perceptions of transformational leadership. The GTL demonstrated strong psychometric properties in this study, with a Cronbach's alpha of 0.89 [88, 89].
B. Organizational Justice Scale (OJS) [90]: This 20-item scale measures perceptions of workplace fairness across three subscales: distributive justice (5 items), procedural justice (6 items), and interactional justice (9 items). Responses are given on a five-point Likert scale, with higher scores indicating a greater perception of fairness. The subscale scores are summed up to generate a total score, with higher scores reflecting stronger perceptions of organizational justice. In this study, the OJS subscales demonstrated excellent internal consistency, with Cronbach's alpha coefficients ranging from 0.88 to 0.92 [91–93].
C. Quality of Nursing Work Life (QNWL) Scale [94, 95]: This 42-item instrument assesses nurses' perceptions of their work–life quality across four dimensions: work–life/home–life balance (7 items), work design (10 items), work context (20 items), and work world (5 items). Responses are scored on a five-point Likert scale, with higher scores indicating a better QWL. The total score is obtained by summing the scores from each dimension. In the current study, Cronbach's alpha values for the subscales ranged from 0.81 to 0.93.
D. Nurse well-being measures:
  1. Emotional Exhaustion Subscale of the Maslach Burnout Inventory (MBI) [96, 97]: This nine-item subscale measures emotional exhaustion on a seven-point frequency scale (0 = never to 6 = everyday). Higher scores indicate greater emotional exhaustion. In this study, the subscale exhibited high reliability, with a Cronbach's alpha of 0.91.
  2. Job Satisfaction Scale (JSS) [98]: This 15-item scale assesses overall job satisfaction using a 7-point Likert scale (1 = strongly disagree to 7 = strongly agree). Higher total scores indicate higher levels of job satisfaction. The JSS demonstrated excellent internal consistency in this study, with Cronbach's alpha of 0.93.
  3. Perceived Stress Scale (PSS-10) [99]: This 10-item scale measures perceived stress levels using a 5-point Likert scale (0 = never to 4 = very often). The total score, ranging from 0 to 40, is calculated by summing the scores of all items, with higher scores reflecting greater levels of perceived stress [94, 95]. Both positive and negative stress perceptions are included, but a total score was used for analysis. The PSS-10 demonstrated acceptable reliability, with Cronbach's alpha of 0.87.

#### 2.7.2. Qualitative Data Collection Tool

A semistructured focus group discussion guide was developed to explore nurses' lived experiences and perceptions of transformational leadership, organizational justice, QWL, and well-being. Experienced moderators conducted the focus group discussions in a safe and nonjudgmental environment. The discussions were recorded on audio, verbatim translated, and analyzed using thematic reflection analysis to identify patterns and themes [63, 92–103].

##### 2.7.2.1. Rigorous Translation and Pilot Testing

To ensure linguistic and cultural adequacy, all quantitative scales have been subject to a strict translation and back-translation process. Independent bilingual experts translated the scales from English to Arabic and then converted them to English. A group of subject experts reviewed the Arabic versions for clarity and content validity. A pilot study of 30 nurses was also conducted to evaluate the feasibility and reliability of the instruments and make small clarification adjustments.

### 2.8. Ethical Approval

The study was approved by the Institute Review Committee (IRB) of Jouf University (3-09-45) and complies with the ethical principles laid down in the Helsinki Declaration. All nurses participating in the study obtained informed consent, which provided detailed information on the purpose, procedures, risks, benefits, and rights of participants. Each nurse received a unique identification code to protect the privacy and confidentiality of the participants, and the data were reported in aggregate form. The data on personal identification were not collected, and potentially identifiable data were securely stored in a password-protected electronic database that was accessible only to researchers. Participation was voluntary, and nurses could withdraw from the study at any time without adverse consequences. The researcher prioritized the participants' well-being and dignity, ensuring that their physical, psychological, and emotional needs were respected.

### 2.9. Procedure

The collection of data for this mixed-method study was carried out in two phases. In the first phase, quantitative data were collected through a cross-sectional survey design. Researchers obtained permission from hospital administrators and nursing departments in each participating hospital to distribute questionnaires to nurses. These questionnaires include the GTL, the OJS, the QNWL Scale, and three measures of nursing well-being (subscale of emotional fatigue, scale of workplace satisfaction, and scale of perceived stress) prepared in English and Arabic to reflect participants' linguistic preferences. The researcher visited each hospital and distributed questionnaires to nurses during their working hours. Nurses were informed of the purpose, the nature of the voluntary study, and measures taken to ensure confidentiality and anonymity. Participants had enough time to complete the questionnaire and were encouraged to ask any questions or concerns they might have had. The completed questionnaires were collected by researchers who checked their accuracy and coherence.

Demographic data were also collected from participants in the survey questionnaire. Population variables include age, gender, marital status, nursing category (e.g. staff nurses, and nurses' managers), and nursing experience. These data were used to describe sample characteristics and to investigate any possible link between demographic variables and key study variables (transformative leadership, organizational justice, working quality, and health of nurses). The inclusion of demographic factors in the data collection process has enabled the researcher better to understand the diversity and representativeness of the sample. The researcher examined the relationships between demographic variables and the main structures of the study and identified any potential differences or patterns that might affect the interpretation of the results among the subgroups. This information is crucial to the development of targeted interventions and strategies that address the specific needs and challenges of various groups of nurses in the Saudi health system. In addition, the collection of demographic data have allowed comparisons between the study sample and the larger nursing population in Saudi Arabia. This comparison has contributed to establishing the generality of the findings and identifying potential limitations or prejudices in sampling. By ensuring that the sample was representative of the wider nursing workforce, researchers could make more confident statements about the applicability of their results in different healthcare environments in the region.

In the second phase, qualitative data were collected through semistructured focus group discussions. The researcher deliberately selected a subset of nurses from those participating in the quantitative survey to ensure a diverse representation in terms of age, gender, experience, and hospital environments. The selected nurses were invited to participate in the discussion of the focus group and obtained their informed consent. The group discussions focused on a quiet and comfortable environment within each hospital and were held at a convenient time for participants. Each focus group was composed of six to eight nurses and moderated by trained researchers. The discussion was led by a sem-structured interview guide that included open questions and probes to explore the experiences and perceptions of nurses regarding transformational leadership, organizational justice, the quality of life at work, and well-being. The discussions were recorded with the participant's permission and subsequently transcribed verbatim for analysis.

Throughout the data collection process, the researcher maintained a reflective journal to record observations, insights, and potential prejudices that might have influenced the study. The researcher also ensured that data collection procedures were consistent across all participating hospitals to minimize any variations that could affect the study results. After completing the two phases of data collection, the researcher carefully reviewed the quantitative and qualitative data to ensure completeness and accuracy. Quantitative data are entered into the statistical software package for analysis, and qualitative data are organized and prepared for thematic analysis. The mixed-method approach enabled the researcher to gain a comprehensive and comprehensive understanding of the relationship between transformational leadership, organizational justice, QWL, and the well-being of nurses. Quantitative data provide measurable evidence of these relationships, while qualitative data provide a more in-depth understanding of nurses' living experiences and the context in which they occur. By integrating the results of the two phases, the researcher sought to develop a more precise and holistic understanding of factors influencing the well-being of nurses in Saudi Arabia. This approach has facilitated the development of evidence-based recommendations for improving nursing leadership, promoting organizational justice, and improving the quality of work, ultimately contributing to the well-being and satisfaction of nursing staff in the region.

### 2.10. Statistical Analysis

Quantitative data were analyzed using IBM SPSS Statistics (Version 28). The preliminary analyses ensured that there were no violations of the assumptions of normality, linearity, multicollinearity, and homoscedasticity. Adequate remediation measures, such as data transformations or robust estimation methods, will be used if violations are detected. Descriptive statistics summarized the demographic and professional characteristics of the sample. The reliability of the scales (GTL Scale, OJS, QNWL Scale, Emotional Exhaustion Subscale, JSS and PSS) was assessed using Cronbach alpha, with values above 0.70, indicating acceptable internal consistency. Pearson correlation analyses examined the bivariate relationships between key variables: transformational leadership, organizational justice dimensions (distributive, procedural, and interactional), QWL dimensions (work–life balance, work design, work context, and work world), emotional exhaustion, job satisfaction, and perceived stress. The hierarchical multiple regression analyses examined the direct and indirect effects of transformational leadership on the well-being of nurses, with organizational justice and the QWL as mediators. Transformational leadership is the first step, followed by organizational justice and the QWL in Step 2. Changes in R2 and standardized coefficients were assessed to determine the predictive power and significance.

The mediation analysis was carried out using the SPSS PROCESS macro (Version 3.5; Hayes, 2018) with bias-corrected bootstrap samples (5000 resamples) to evaluate the effects of the mediator of organizational justice and the quality of work on the relationship between transformational leadership and the results of well-being. Indirect effects were considered significant when the confidence intervals were excluded from zero. Model adaptability was evaluated using indices such as the comparison fit index (CFI), the Tucker–Lewis Index (TLI), and the approximation error of the root square (RMSEA). For qualitative data, audio recordings from the focus groups were transcribed verbatim and analyzed using thematic analysis [50]. An inductive semantic approach was used to identify meaning patterns in the whole set. The original codes were created and iteratively reviewed, then organized into themes that reflected key concepts related to transformational leadership, organizational justice, the QWL, and the well-being of nurses. Furthermore, the potential influence of demographic and professional characteristics on the key study variables was investigated with the help of multivariate analysis of variance (MANOVA) and moderate regression analysis.

These analyses provide an overview of how factors such as age, gender, nursing category, and years of experience affect the relationships under study. Effect sizes and confidence intervals were reported for all important results in order to provide standardized measurements of practical significance and facilitate comparison with other studies. The integration of quantitative and qualitative results took place during the interpretation phase and the results were synthesized to investigate convergence, divergence, and complementarity. This mixed-method approach has provided a comprehensive understanding of the relationship between transformational leadership, organizational justice, QWL, and the well-being of nurses. The findings were discussed in relation to the theoretical and practical implications for nursing, leadership development, and health policy. The analysis followed robust, contemporary techniques, checked statistical hypotheses, reported the effects of their magnitude, and maintained an objective, critical position throughout interpretation, considering limitations and alternative explanations.

## 3. Results

The results of this mixed-method study provide a comprehensive understanding of the relationship between the transformational leadership of Saudi medical care, organizational justice, work quality, and the well-being of nurses. Quantitative results from a sample of 580 nurses in five hospitals in the Jouf region have highlighted the direct and indirect effects of transformational leadership on the well-being of nurses, with organizational justice and the quality of life of the working person acting as key mediators. The qualitative insights of the discussions of the focus groups with 25 nurses provided valuable perspectives on nurses' lives and perceptions of how transformational leadership, fair treatment, and supportive working environments enhance their well-being. The integration of these quantitative and qualitative results gives a detailed overview of the complex interactions between leadership, organizational factors, and the well-being of nurses, emphasizing the importance of promoting transformative leadership practices, promoting organizational justice, and improving the QWL to support the well-being of nurses.

Table 1 presents a comprehensive summary of the demographic and professional characteristics of the sample consisting of 580 nurses. The Table 1 gives a detailed breakdown of each variable, including age, sex, marriage status, nursing category, nursing experience, highest education qualifications, employment status, and shift work. The average age of participants was 35.6 years (SD = 8.2), with the majority being women (79.3%), married (65.5%), and holding nursing positions (72.4%). On average, participants had 10.5 years (SD = 7.8) of nursing experience, and most held a bachelor's degree in nursing (60.4%). Most nurses worked full-time (94.8%) and worked only on day shifts (55.2%). This detailed Table 1 effectively summarizes the main characteristics of the sample and enables readers to understand the composition of the participants better and provides a context for the interpretation of the results of the study.

Table 2 shows the reliability analysis of the measurement scale used in the study, indicating the internal coherence of each scale and its subscale. The GTL Scale has seven subscales, and the Cronbach alpha values range from 0.86 to 0.92. Similarly, the OJS and its three subscales are very reliable, and the Cronbach alpha coefficients are 0.89–0.94. The QNWL Scale and its four subscales also show strong internal coherence, with the alpha values of Cronbach between 0.87 and 0.95. The MBI subscale of emotional exhaustion is higher than Cronbach's 0.93 alpha. At the same time, the JSS and the PSS are reliable in the 0.96 and 0.91 alpha coefficients of Cronbach. These high Cronbach alpha values show that the components of each scale are consistent in measuring the same basic structure, indicating the reliability and credibility of the measuring instruments used in this study.

The Pearson correlation matrix in Table 3 provides valuable information about the relationships between key research variables. Transformational leadership shows significant positive correlations with organizational justice dimensions, QWL dimensions, and employment satisfaction while showing significant negative correlations with emotional exhaustion and perceived stress. Organizational justice and the QWL dimensions also show strong positive correlations with each other and with job satisfaction while exhibiting significant negative correlations with emotional exhaustion and perceived stress. Emotional fatigue and perceived stress have a strong positive correlation and are negatively associated with job satisfaction. These findings suggest that transformational leadership, organizational justice, and the QWL are positively related to the satisfaction of the workplace but negatively related to emotional exhaustion and perceived stress. These results are the basis for further analysis to explore the direct and indirect effects of transformational leadership on nurses' health outcomes.

Hierarchical multiple regression analyses in Table 4 illustrate transformational leadership's direct and indirect effects on emotional exhaustion, job satisfaction, and perceived stress, with organizational justice and QWL dimensions serving as mediator variables. In Step 1, transformational leadership significantly predicts all three dependent variables, showing negative associations with emotional exhaustion (*β* = −0.48) and perceived stress (*β* = −0.46) and a positive association with job satisfaction (*β* = 0.53). The inclusion of organizational justice and QWL dimensions in Step 2 significantly enhances the explanatory power of the models, as indicated by the increased *R*^2^ values and significant ΔF statistics. The standardized regression coefficients of Step 2 show that transformational leadership, organizational justice, and QWL dimensions are all important predictors of emotional fatigue, job satisfaction, and perceived stress, and the strongest effects are observed in the context of work and the design of work. These results suggest that transformational leadership directly impacts nurses' well-being outcomes and indirectly influences them through its effects on organizational justice and perceptions of QWL.

Table 5 presents the results of the mediation analysis examining the direct, indirect, and total effects of transformational leadership on nurse well-being, operationalized as job satisfaction and perceived stress. The analysis considers the mediating roles of organizational justice and QWL. For job satisfaction, transformational leadership exerts both a significant direct effect (estimate = 0.15, *p* < 0.001) and strong indirect effects through organizational justice (estimate = 0.38, *p* < 0.001) and QWL (estimate = 0.38, *p* < 0.001). The total effect of transformational leadership on job satisfaction is substantial (estimate = 0.53, *p* < 0.001), suggesting that both mediators play critical roles in enhancing job satisfaction among nurses. For perceived stress, transformational leadership demonstrates a significant negative direct effect (estimate = −0.13, *p*=0.005), indicating that effective leadership reduces perceived stress. Furthermore, the analysis shows significant indirect effects through organizational justice (estimate = −0.33, *p* < 0.001) and QWL (estimate = −0.33, *p* < 0.001). The total effect (estimate = −0.46, *p* < 0.001) highlights the combined impact of direct leadership influence and the mediating factors in reducing perceived stress. Overall, the findings indicate that both organizational justice and QWL significantly mediate the relationship between transformational leadership and nurse well-being. These results support the study's theoretical framework and suggest that improving leadership practices and work environment conditions can enhance job satisfaction and reduce stress among nurses.

The thematic analysis presented in Table 6 highlights the key themes and subthemes that emerged from the qualitative focus group discussions, providing valuable information on nurses' experiences and perceptions of transformational leadership, organizational justice, QWL, and well-being. The five main identified themes include transformational leadership, organizational justice, QWL, nurse well-being, and the impact of leadership on well-being. Each theme is divided into subthemes, with representative quotes from participants illustrating the essence of each subtheme. The Table 6 effectively summarizes the rich qualitative data, demonstrating the importance of transformational leadership behaviors, fair treatment, and a supportive work environment in promoting nurse well-being. The inclusion of direct quotes adds depth and authenticity to the findings, allowing readers to understand better the lived experiences of the nurses in the study. This thematic analysis complements the quantitative results, offering a more comprehensive understanding of the complex relationships between leadership, organizational factors, and nurse well-being.

The thematic analysis explored not only positive perceptions and experiences but also deeper meanings and underlying challenges nurses face in their work environment. While the themes predominantly reflect positive experiences, instances of dissatisfaction or unmet expectations were also discussed, such as perceived inequities in procedural justice or challenges in achieving work–life balance.

Table 7 presents an integration of the quantitative and qualitative findings on the relationships between transformational leadership, organizational justice, QWL, and nurse well-being. The Table 7 highlights how transformational leadership significantly improves nurse well-being, reducing emotional exhaustion, increasing job satisfaction, and lowering perceived stress. The mediating roles of organizational justice and QWL are clearly established, with indirect effects ranging from −0.34 to 0.38 (*p* < 0.001) for organizational justice and from −0.33 to 0.38 (*p* < 0.001) for QWL. These results indicate that both mediators significantly influence the relationship between transformational leadership and well-being outcomes. Nurses emphasized the importance of fair treatment, work–life balance, and professional development, which align with the quantitative findings. For policy and practice, this Table 7 reinforces the need for healthcare organizations to prioritize leadership development, promote fairness in decision-making, and improve nurses' work environments through targeted interventions. A holistic approach addressing leadership, organizational justice, and QWL is essential for enhancing nurse well-being and reducing burnout.

## 4. Discussion

The primary objective of this mixed methods' study was to investigate the impact of transformational leadership on nurse well-being within the context of Saudi Arabian healthcare. The study aimed to address the central research question: How does transformational leadership affect nurse well-being, and what roles do organizational justice and QWL play as mediators? By employing a sequential explanatory design, the study sought to provide a comprehensive understanding of the complex interplay between leadership, organizational factors, and nurse well-being outcomes. The integration of quantitative and qualitative data allowed for a robust examination of the direct and indirect effects of transformational leadership on nurses' emotional exhaustion, job satisfaction, and perceived stress while also illuminating the lived experiences and perceptions of nurses in their work environment.

The quantitative phase of the study yielded several key findings that shed light on the relationships between transformational leadership, organizational justice, QWL, and nurse well-being. Hierarchical multiple regression analyses revealed significant direct effects of transformational leadership on all three well-being outcomes. Specifically, higher levels of transformational leadership have been associated with reduced emotional fatigue, greater satisfaction with work, and reduced perceived stress among nurses. These results demonstrate the significant impact that transformative nurses can have on reducing burnout, promoting workplace satisfaction, and reducing the stress of the nursing workforce. Furthermore, mediation analysis provides evidence of the important indirect impact of transformational leadership on the well-being of nurses through the quality of organizational justice and work–life aspects. The results showed that transformational leadership influences nurses' perceptions of fairness in the workplace (organizational justice) and their general work experience (QWL), which in turn contributes to improving the results of well-being.

The qualitative phase of the study, conducted through focus group discussions, provided rich insights into nurses' experiences and perceptions of transformational leadership, organizational justice, QWL, and well-being. Thematic analysis revealed several key themes that complemented and expanded upon the quantitative findings. Nurses described specific transformational leadership behaviors, such as inspirational motivation, individualized consideration, intellectual stimulation, and idealized influence, as crucial for their well-being. They emphasized how transformational leaders, who communicate a clear vision, provide personalized support, encourage innovative thinking, and lead by example, create a positive work environment that fosters their well-being. Furthermore, nurses highlighted the importance of organizational justice in their work lives. They discussed how fair resource allocation, transparent decision-making processes, and respectful treatment from supervisors contribute to a sense of justice and equity in the workplace, which positively influences their well-being. Nurses also emphasized the significance of QWL factors, such as work–life balance, professional development opportunities, and a supportive work environment, in promoting their overall well-being. The qualitative conclusions provide a context and depth to the quantitative results and illustrate the complex mechanisms that affect the well-being of nurses through the interaction of transformational leadership, organizational justice, and QWL. Although the focus group discussions highlighted primarily positive perceptions of transformational leadership, organizational justice, and QWL, nuances of negative experiences, such as resource constraints and occasional communication breakdowns, were also reported. These were integrated into the analysis to provide a balanced interpretation.

The significant direct effects of transformational leadership on reducing emotional exhaustion and perceived stress and increasing job satisfaction underscore the pivotal role that transformational nurse leaders play in promoting nurse well-being. these findings are consistent with the proposal of transformation leadership theory, which says that transformation leaders inspire, motivate, and support their followers, resulting in a positive result associated with work [12, 43, 64]. The qualitative data further support these findings by illustrating specific behaviors of transformational leaders that contribute to nurse well-being. Nurses described how transformational leaders who provide individualized support, encourage professional growth, and foster a positive work environment create conditions conducive to their emotional, mental, and physical well-being. These findings highlight the importance of cultivating transformational leadership skills among nurse managers to mitigate burnout, enhance job satisfaction, and reduce stress in the nursing workforce.

The mediation analysis results revealed that organizational justice dimensions (distributive, procedural, and interactional justice) partially mediate the relationship between transformational leadership and nurse well-being. This suggests that transformational leaders promote perceptions of fairness in the workplace, which, in turn, positively influences nurses' well-being. Distributive justice significantly contributed to reducing emotional exhaustion and perceived stress by fostering a sense of equity and fairness in resource allocation. Procedural justice was strongly linked to increased job satisfaction, as transparent and inclusive decision-making processes instilled a sense of organizational trust and empowerment. Interactional justice played a pivotal role in mitigating perceived stress and enhancing emotional well-being by ensuring respectful, considerate treatment from supervisors. These findings highlight the nuanced roles of each dimension in shaping different aspects of nurse well-being.

These predominantly positive perceptions of organizational justice among nurses may reflect high levels of organizational identification within the sample. Organizational identification strengthens employees' alignment with organizational values, thereby enhancing their perceptions of fairness. Previous research has demonstrated that employees with strong identification tend to report higher perceptions of distributive, procedural, and interactional justice, as these align with their intrinsic commitment to the organization [104]. Furthermore, ethical leadership practices that reinforce justice have been shown to bolster employees' organizational identification, creating a reciprocal dynamic that promotes trust and fairness in healthcare settings [105]. These findings suggest that organizational identification serves as an important contextual factor influencing perceptions of justice and its subsequent effects on nurse well-being. These findings highlight the nuanced roles of each dimension in shaping different aspects of nurse well-being.

The qualitative data provided rich examples of how nurses experience fairness under transformational leadership, such as equitable resource allocation, transparent decision-making processes, and respectful interpersonal treatment. Nurses emphasized how these experiences of justice contribute to their sense of well-being and motivation at work. These findings underscore the importance of fostering organizational justice in healthcare settings, as it serves as a critical pathway through which transformational leadership can enhance nurse well-being.

The study also highlighted the mediating role of QWL dimensions (work–life balance, work design, work context, and work world) in the relationship between transformational leadership and nurse well-being. Transformational leaders prioritizing and supporting these aspects of work life create a more positive and fulfilling work experience for nurses, leading to improved well-being outcomes. The qualitative insights provided vivid examples of how transformational leaders' actions, such as promoting flexibility, providing growth opportunities, and fostering a collaborative work environment, enhance nurses' QWL and, consequently, their well-being. These findings emphasize the need for healthcare organizations to focus on improving the QWL for nurses, as it is a key mechanism through which transformational leadership can positively impact nurse well-being.

The findings of this study are supported by recent research that highlights the positive impact of transformational leadership on employee well-being [43, 48, 94, 96] and found that transformational leadership is associated with reduced burnout and increased job satisfaction among nurses. These studies corroborate the current study's findings on the direct effects of transformational leadership on emotional exhaustion, job satisfaction, and perceived stress. However, there are also studies that present contradictory findings [89, 104, 105] that reported that while transformational leadership positively impacts job satisfaction, its effect on emotional exhaustion was not significant. This discrepancy may be due to different organizational contexts and cultural factors that influence how transformational leadership is perceived and enacted. Future research should explore these variations to provide a more nuanced understanding of the conditions under which transformational leadership is most effective.

## 5. Theoretical Implications

The findings of the study support and expand the theory of transformational leadership in the context of nursing care. The major direct and indirect effects of transformational leadership on the results of nurse well-being demonstrate the applicability and relevance of the theory in understanding the influence of leadership on the well-being of employees in healthcare settings. The study provides empirical evidence for the positive influence of transformational leadership behaviors on nurses' emotional, mental, and physical well-being, thus expanding the theory's explanatory power. Moreover, the study highlights the specific transformational leadership behaviors that are most salient in promoting nurse well-being, such as individualized consideration, inspirational motivation, and idealized influence. These findings contribute to the refinement and contextual application of transformational leadership theory in the nursing profession.

The findings are consistent with the theory of social exchange, where transformational leadership fosters reciprocal exchanges by promoting fairness and improving work–life quality. Nurses, in turn, respond to these positive exchanges with higher job satisfaction and reduced stress, reinforcing the importance of reciprocal relationships in enhancing nurse well-being. These findings align with the principles of social exchange theory, which posits that fair and respectful treatment fosters positive reciprocal relationships between employees and their organizations. Nurses who perceive higher levels of organizational justice are likely to experience reduced emotional exhaustion and stress while reciprocating with higher job satisfaction and commitment. This reinforces the notion that organizational justice acts as the key mechanism through which transformational leadership influences nurse well-being, as fairness perceptions strengthen the social exchange dynamic between nurses and their workplace. The results also highlight the importance of the theory of social exchange in understanding the relationship between transformational leadership, organizational justice, QWL, and the well-being of nurses. Social exchange theory posits that when employees perceive their leaders as supportive and fair, they are more likely to reciprocate with positive attitudes and behaviors. The study found that nurses who viewed their leaders as transformational reported a high level of organizational justice and a high quality of life, which in turn contributed to enhancing the outcome of happiness. These findings suggest that transformational leadership creates a positive social exchange dynamic wherein nurses feel valued, supported, and treated fairly. This leads them to reciprocate with increased job satisfaction, reduced emotional exhaustion, and lower perceived stress. The study thus integrates transformational leadership theory with social exchange theory, providing a more comprehensive understanding of the mechanisms underlying the relationship between leadership and employee well-being in the nursing context.

## 6. Practical Implications

The study's findings underscore the critical importance of investing in transformational leadership training programs for nurse managers. Healthcare organizations should prioritize the development of transformational leadership skills among their nursing leadership to create a supportive and empowering work environment that promotes nurse well-being. Such training programs should focus on enhancing nurse managers' abilities to inspire and motivate their staff, provide individualized support and consideration, encourage intellectual stimulation, and lead by example. By equipping nurse managers with these transformational leadership competencies, healthcare organizations can foster a leadership culture that prioritizes and supports the well-being of the nursing workforce, ultimately leading to better patient care outcomes and organizational performance.

The study's results also highlight the need for healthcare organizations to implement policies and practices that promote organizational justice and QWL. To amplify the positive effects of transformational leadership on nurse well-being, organizations should focus on creating fair and transparent systems for resource allocation, decision-making, and performance evaluation. This can include establishing clear and consistent criteria for promotions, providing regular feedback and communication channels, and ensuring that nurses have a voice in organizational processes that affect their work lives. In addition, policies that support work–life balance, such as flexible scheduling options, adequate staffing levels, and opportunities for professional development, can enhance nurses' QWL and contribute to their overall well-being. By institutionalizing these policies and practices, healthcare organizations can create a work environment that complements and reinforces the positive impact of transformational leadership on nurse well-being.

The findings of this study suggest that a comprehensive wellness program that addresses leadership, fairness, and work–life quality in healthcare settings can be an effective approach to promoting nurse well-being. Rather than focusing on isolated interventions, healthcare organizations should adopt a holistic perspective that recognizes the interconnectedness of these factors in shaping nurses' well-being. A comprehensive wellness program could include transformational leadership training for nurse managers, workshops on effective communication and conflict resolution, stress management and resilience-building resources for nurses, and initiatives to foster a culture of respect, collaboration, and work–life balance. By taking a multifaceted approach that targets multiple levels of the organization, healthcare institutions can create a work environment that supports and enhances nurses' physical, emotional, and professional well-being, ultimately leading to better outcomes for both nurses and patients.

## 7. Limitations

Although this study provides valuable insights into the relationship between transformational leadership, organizational justice, the quality of work, and the well-being of nurses, it is important to recognize their limitations. The cross-sectional nature of the quantitative data precludes the establishment of causal relationships between the study variables. Although the mediation analyses suggest potential causal pathways, the study's design cannot definitively confirm the directionality of these relationships. In addition, the self-report nature of the survey measures may introduce common method bias, as nurses' responses could be influenced by social desirability or other subjective factors. To address these limitations, future research should employ longitudinal designs that can track changes in the study variables over time and establish temporal precedence. Incorporating objective measures of well-being, such as physiological indicators or absenteeism rates, could also help to corroborate the self-reported findings and reduce the potential for common method bias.

Another limitation of the study is the potentially limited generalizability of the findings to other cultural and healthcare contexts. While the study sample was diverse and representative of nurses in the Jouf region of Saudi Arabia, it is important to consider the unique cultural, social, and organizational factors that may influence the relationships between transformational leadership, organizational justice, QWL, and nurse well-being in this specific context. The Saudi Arabian healthcare system has its own distinct characteristics, such as a high reliance on expatriate nurses, gender segregation in the workforce, and cultural values that emphasize collectivism and hierarchical relationships. These contextual factors may shape the way transformational leadership is enacted and experienced by nurses, as well as the relative importance of organizational justice and QWL in influencing their well-being. Therefore, caution should be exercised when applying the study's findings to other cultural or healthcare settings, as the dynamics between the study variables may vary. Future research should aim to replicate this study in diverse contexts to assess the generalizability of the findings and identify potential boundary conditions or cultural moderators.

## 8. Future Research Directions

In order to establish causal relationships between transformational leadership, organizational justice, the QWL, and the well-being of nurses, future research should use longitudinal research designs. By collecting data at multiple time points, researchers can examine how changes in transformational leadership, perceptions of fairness, and work–life quality influence nurses' well-being outcomes over time. Longitudinal studies can also help to identify potential reciprocal relationships between the study variables, such as the possibility that nurses with higher levels of well-being may be more likely to perceive their leaders as transformational or their work environment as fair and supportive. In addition, longitudinal designs can shed light on the long-term effects of transformational leadership and organizational factors on nurses' job attitudes, performance, and retention, providing valuable insights for healthcare organizations seeking to build and sustain a healthy, engaged nursing workforce.

Future research should also explore other potential mediators and moderators in the relationship between transformational leadership and nurse well-being. While this study focused on organizational justice and QWL as key mediating mechanisms, there may be additional factors that help to explain how transformational leadership influences nurses' well-being. For example, psychological capital (encompassing self-efficacy, hope, resilience, and optimism) has been shown to mediate the relationship between transformational leadership and employee well-being in other occupational contexts [106]. Investigating the role of psychological capital in the nursing context could provide a more comprehensive understanding of the psychological processes underlying the effects of transformational leadership on nurse well-being. Similarly, exploring the moderating role of individual characteristics, such as personality traits, coping styles, or cultural values, could help to identify boundary conditions for the effectiveness of transformational leadership in promoting nurse well-being. By examining a broader range of mediators and moderators, future research can refine and extend the theoretical framework proposed in this study, leading to a more nuanced understanding of the complex interplay between leadership, Organizational factors, and nurse well-being.

Future research should aim to replicate this study in diverse healthcare settings and cultural contexts to assess the generalizability of the study's findings and identify potential cultural differences in the relationships between transformational leadership, organizational justice, QWL, and nurse well-being. Conducting cross-cultural comparisons can provide valuable insights into the universality or cultural specificity of the observed relationships, as well as the relative importance of different transformational leadership behaviors, organizational justice dimensions, and QWL factors in shaping nurse well-being. For example, future studies could compare the findings from Saudi Arabia with those from other Middle Eastern countries, Western countries, or Asian countries to explore how cultural values, societal norms, and healthcare system characteristics may influence the dynamics between the study variables. In addition, replicating the study in different types of healthcare organizations (e.g., public vs. private and acute care vs. long-term care) and with different nursing populations (e.g., novice vs. experienced nurses and specialty vs. general practice nurses) can help to identify potential boundary conditions and refine the theoretical model better to capture the diversity of nursing experiences and contexts.

## 9. Conclusion

This mixed-method study provides a comprehensive understanding of the impact of transformational leadership on nurses' health, bringing an important contribution to nursing research and practice, and serves as an important mediation mechanism for organizational justice and work–life quality. The integration of quantitative and qualitative findings offers a robust and nuanced perspective on the complex interplay between leadership, organizational factors, and nurse well-being outcomes, highlighting the critical role of transformational nurse leaders in creating positive work environments that promote fairness, support work–life balance, and enhance the overall well-being of the nursing workforce. The study's findings have important implications for healthcare organizations seeking to promote nurse well-being and optimize organizational performance. By investing in transformational leadership development programs, implementing policies that foster organizational justice and QWL, and adopting a holistic approach to nurse well-being, healthcare institutions can create a work environment that supports and empowers nurses, ultimately leading to better patient care outcomes and organizational effectiveness.

As the nursing profession continues to face significant challenges, including high levels of burnout, turnover, and a global shortage of nurses, the insights from this study provide a timely and relevant framework for addressing these pressing issues. By focusing on the well-being of their nursing staff and cultivating a transformational leadership at all levels of the organization, health institutions can build a resilient, committed, and flourishing nursing staff that is better able to meet the complex requirements of 21st-century healthcare. Finally, the study highlighted the importance of transformative leadership, organizational justice, and the QWL to the development of the well-being of nurses. It offers a compelling call to action for healthcare organizations to prioritize these factors in their efforts to support and empower their nursing workforce. By doing so, they can not only enhance the well-being of individual nurses but also contribute to the broader goal of building a sustainable, high-quality healthcare system that meets the needs of patients, providers, and society.

## Figures and Tables

**Table 1:** The demographic and professional characteristics of the sample (*N* = 580).

Variable	*N*	%
Age (years)		
20–29	120	20.7%
30–39	280	48.3%
40–49	150	25.9%
50 and above	30	5.1%
Mean (SD)	35.6 (8.2)	
Gender		
Male	120	20.7%
Female	460	79.3%
Marital status		
Single	180	31.0%
Married	380	65.5%
Divorced	15	2.6%
Widowed	5	0.9%
Nursing category		
Staff nurse	420	72.4%
Nurse manager	100	17.2%
Nurse educator	60	10.4%
Years of nursing experience		
1–5	150	25.9%
6–10	200	34.5%
11–15	120	20.7%
16–20	70	12.0%
More than 20	40	6.9%
Mean (SD)	10.5 (7.8)	
Highest educational qualification		
Diploma in nursing	180	31.0%
Bachelor of Science in Nursing	350	60.4%
Master of Science in Nursing	45	7.8%
Doctorate in Nursing	5	0.8%
Employment status		
Full-time	550	94.8%
Part-time	30	5.2%
Shift work		
Day shift only	320	55.2%
Night shift only	80	13.8%
Rotating shifts	180	31.0%

**Table 2:** Reliability analysis of the measurement scales.

Scale	Number of items	Cronbach's alpha
Global Transformational Leadership (GTL)	7	0.92
Vision	3	0.88
Staff development	3	0.90
Supportive leadership	3	0.87
Empowerment	3	0.89
Innovative thinking	3	0.91
Lead by example	4	0.86
Charisma	3	0.90
Organizational Justice (OJS)	20	0.94
Distributive justice	5	0.89
Procedural justice	6	0.91
Interactional justice	9	0.93
Quality of Nursing Work Life (QNWL)	42	0.95
Work–life/home–life	7	0.88
Work design	10	0.92
Work context	20	0.94
Work world	5	0.87
Maslach Burnout Inventory (MBI)		
Emotional Exhaustion	9	0.93
Job Satisfaction Scale (JSS)	36	0.96
Perceived Stress Scale (PSS)	10	0.91

**Table 3:** Pearson correlation matrix of key study variables.

Variables	1	2	3	4	5	6	7	8	9	10
1. Transformational leadership	1									
2. Distributive justice	0.56⁣^∗∗^	1								
3. Procedural justice	0.62⁣^∗∗^	0.71⁣^∗∗^	1							
4. Interactional justice	0.59⁣^∗∗^	0.68⁣^∗∗^	0.73⁣^∗∗^	1						
5. Work–life/home–life	0.45⁣^∗∗^	0.52⁣^∗∗^	0.57⁣^∗∗^	0.55⁣^∗∗^	1					
6. Work design	0.51⁣^∗∗^	0.58⁣^∗∗^	0.63⁣^∗∗^	0.60⁣^∗∗^	0.66⁣^∗∗^	1				
7. Work context	0.54⁣^∗∗^	0.61⁣^∗∗^	0.67⁣^∗∗^	0.64⁣^∗∗^	0.70⁣^∗∗^	0.75⁣^∗∗^	1			
8. Work world	0.42⁣^∗∗^	0.49⁣^∗∗^	0.53⁣^∗∗^	0.51⁣^∗∗^	0.56⁣^∗∗^	0.60⁣^∗∗^	0.64⁣^∗∗^	1		
9. Emotional exhaustion	−0.48⁣^∗∗^	−0.55⁣^∗∗^	−0.60⁣^∗∗^	−0.57⁣^∗∗^	−0.62⁣^∗∗^	−0.67⁣^∗∗^	−0.71⁣^∗∗^	−0.54⁣^∗∗^	1	
10. Job satisfaction	0.53⁣^∗∗^	0.60⁣^∗∗^	0.66⁣^∗∗^	0.63⁣^∗∗^	0.69⁣^∗∗^	0.74⁣^∗∗^	0.78⁣^∗∗^	0.59⁣^∗∗^	−0.76⁣^∗∗^	1
11. Perceived stress	−0.46⁣^∗∗^	−0.53⁣^∗∗^	−0.58⁣^∗∗^	−0.55⁣^∗∗^	−0.60⁣^∗∗^	−0.65⁣^∗∗^	−0.69⁣^∗∗^	−0.52⁣^∗∗^	0.73⁣^∗∗^	−0.71⁣^∗∗^

⁣^∗∗^*p* < 0.01.

**Table 4:** Hierarchical multiple regression analyses for emotional exhaustion, job satisfaction, and perceived stress.

Predictor	Emotional exhaustion			Job satisfaction			Perceived stress		
	Step 1	Step 2	95% CI	Step 1	Step 2	95% CI	Step 1	Step 2	95% CI
	*β* (*t*)	*β* (*t*)		*β* (*t*)	*β* (*t*)		*β* (*t*)	*β* (*t*)	
Transformational leadership	−0.48⁣^∗∗∗^ (−12.98)	−0.14⁣^∗∗^ (−3.24)	[−0.23, −0.06]	0.53⁣^∗∗∗^ (14.83)	0.15⁣^∗∗^ (3.63)	[0.07, 0.23]	−0.46⁣^∗∗∗^ (−12.36)	−0.13⁣^∗∗^ (−2.97)	[−0.22, −0.05]
Distributive justice		−0.12⁣^∗^ (−2.34)	[−0.22, −0.02]		0.13⁣^∗∗^ (2.66)	[0.03, 0.22]		−0.11⁣^∗^ (−2.21)	[−0.21, −0.01]
Procedural justice		−0.17⁣^∗∗^ (−2.99)	[−0.28, −0.06]		0.19⁣^∗∗∗^ (3.55)	[0.09, 0.30]		−0.16⁣^∗∗^ (−2.87)	[−0.27, −0.05]
Interactional justice		−0.10⁣^∗^ (−2.07)	[−0.19, −0.01]		0.11⁣^∗^ (2.37)	[0.02, 0.20]		−0.09⁣^∗^ (−1.98)	[−0.18, 0.00]
Work–life/home–life		−0.14⁣^∗∗^ (−3.11)	[−0.23, −0.05]		0.16⁣^∗∗∗^ (3.70)	[0.08, 0.25]		−0.13⁣^∗∗^ (−2.93)	[−0.22, −0.04]
Work design		−0.19⁣^∗∗∗^ (−3.81)	[−0.29, −0.09]		0.22⁣^∗∗∗^ (4.66)	[0.13, 0.31]		−0.18⁣^∗∗∗^ (−3.67)	[−0.28, −0.08]
Work context		−0.24⁣^∗∗∗^ (−4.42)	[−0.35, −0.13]		0.27⁣^∗∗∗^ (5.28)	[0.17, 0.37]		−0.23⁣^∗∗∗^ (−4.26)	[−0.34, −0.12]
Work world		−0.08⁣^∗^ (−2.01)	[−0.16, −0.01]		0.09⁣^∗^ (2.32)	[0.01, 0.16]		−0.07⁣^∗^ (−1.96)	[−0.15, 0.00]
*R* ^2^	0.23	0.56		0.28	0.63		0.21	0.54	
Adjusted *R*^2^	0.23	0.55		0.28	0.62		0.21	0.53	
*F*	168.33⁣^∗∗∗^	90.77⁣^∗∗∗^		220.05⁣^∗∗∗^	118.83⁣^∗∗∗^		152.72⁣^∗∗∗^	83.65⁣^∗∗∗^	
Δ*R*^2^		0.33			0.35			0.33	
Δ*F*		61.36⁣^∗∗∗^			74.79⁣^∗∗∗^			57.41⁣^∗∗∗^	

⁣^∗^*p* < 0.05.

⁣^∗∗^*p* < 0.01.

⁣^∗∗∗^*p* < 0.00.

**Table 5:** Mediation analysis: direct, indirect, and total effects of transformational leadership on nurse well-being outcomes.

Outcome	Effect	Path	Estimate	95% CI	*p* value
Job satisfaction	Direct	Transformational leadership ⟶ nurse well-being	0.15	[0.06, 0.24]	0.001
	Indirect	Transformational leadership ⟶ organizational justice ⟶ nurse well-being	0.38	[0.30, 0.46]	< 0.001
	Indirect	Transformational leadership ⟶ quality of work–life ⟶ nurse well-being	0.38	[0.30, 0.46]	< 0.001
	Total	Transformational leadership ⟶ nurse well-being	0.53	[0.45, 0.61]	< 0.001

Perceived stress	Direct	Transformational leadership ⟶ nurse well-being	−0.13	[−0.22, −0.04]	0.005
	Indirect	Transformational leadership ⟶ organizational justice ⟶ nurse well-being	−0.33	[−0.41, −0.25]	< 0.001
	Indirect	Transformational leadership ⟶ quality of work–life ⟶ nurse well-being	−0.33	[−0.41, −0.25]	< 0.001
	Total	Transformational leadership ⟶ nurse well-being	−0.46	[−0.54, −0.38]	< 0.001

*Note:* The mediation analysis results indicate significant direct, indirect, and total effects of transformational leadership on nurse well-being outcomes, highlighting the mediating roles of organizational justice and quality of work life (all effects are significant at *p* < 0.001). Estimates and confidence intervals are based on bias-corrected bootstrap samples (5000 resamples).

**Table 6:** Thematic analysis of focus group discussions: nurses' experiences and perceptions.

Themes	Subthemes	Representative quotes
1. Transformational leadership	1.1. Inspirational motivation	“My nurse manager always encourages us to strive for excellence and sets a clear vision for our team”
	1.2. Individualized consideration	“Our leader takes the time to understand each nurse's strengths and weaknesses and provides personalized support”
	1.3. Intellectual stimulation	“We are encouraged to think critically and find innovative solutions to problems.”
	1.4. Idealized influence	“Our nurse managers lead by example and demonstrate a strong commitment to patient care and staff well-being”

2. Organizational justice	2.1. Distributive justice	“Resources and fair rewards are allocated based on our contributions and efforts”
	2.2. Procedural justice	“Decision-making processes are transparent, and we have opportunities to provide input and feedback”
	2.3. Interactional justice	“Our managers treat us with respect and dignity, providing timely and clear information”

3. Quality of work life	3.1. Work–life balance	“The hospital offers a flexible schedule and supports our efforts to provide a healthy work–life balance”
	3.2. Professional development opportunities	“We have an ongoing training and education program that helps us improve our knowledge and skills”
	3.3. Supportive work environment	“My colleagues and I support each other and create a positive collaborative working environment”

4. Nurse well-being	4.1 Emotional well-being	“When I feel emotionally drained, my boss offers support and encourages me to take breaks when needed.”
	4.2. Physical well-being	“The hospital invests in equipment and promotes safer patient handling to reduce the risk of bodily harm”
	4.3. Professional well-being	“I feel a sense of accomplishment and pride in my work, knowing that I am making a positive difference in patients' lives”

5. Impact of leadership on well-being	5.1. Positive influence of transformational leadership	“Our nurse manager's transformational leadership style has created a supportive and empowering work environment that enhances our well-being”
	5.2. Importance of organizational justice and quality of work life	“Feeling fairly treated and having a good work–life positively affects our overall well-being and ability to provide high-quality patient care”

**Table 7:** The integration of quantitative and qualitative conclusions: transformational leadership, organizational justice, and quality of work life and health of nurses.

Key findings	Quantitative results	Qualitative themes	Implications for practice and policy
1. Transformational leadership and nurse well-being	- Transformational leadership is significantly associated with reduced emotional exhaustion (*β* = −0.48, *p* < 0.001), increased job satisfaction (*β* = 0.53, *p* < 0.001), and lower perceived stress (*β* = −0.46, *p* < 0.001) among nurses	- Nurses described transformational leadership behaviors, such as inspirational motivation, individualized consideration, intellectual stimulation, and idealized influence, as crucial for their well-being	- Healthcare organizations should prioritize the development and training of transformational leadership skills among nurse managers to promote nurse well-being and reduce burnout
2. Mediating role of organizational justice	- Organizational justice dimensions (distributive, procedural, and interactional) significantly mediate the relationship between transformational leadership and nurse well-being outcomes (indirect effects: −0.34 to 0.38, *p* < 0.001)	- Nurses emphasized the importance of fair resource allocation, transparent decision-making processes, and respectful treatment from supervisors to promote a sense of justice and well-being	- Policies and practices that foster organizational justice, such as fair performance appraisals, consistent application of rules, and open communication channels, should be implemented to enhance nurse well-being
3. Mediating role of quality of work life	- Quality of work life dimensions (work–life/home–life, work design, work context, and work world) significantly mediate the relationship between transformational leadership and nurse well-being outcomes (indirect effects: −0.33 to 0.38, *p* < 0.001)	- Nurses identified work–life balance, professional development opportunities, and a supportive work environment as key aspects of quality of work life that contribute to their well-being	- Healthcare organizations should invest in initiatives that improve nurses' quality of work life, such as flexible scheduling, career advancement programs, and team-building activities, to maximize leadership impact
4. Importance of leadership and organizational factors	- Transformational leadership, organizational justice, and quality of work life collectively explain a significant portion of the variance in nurse well-being outcomes (*R*^2^ = 0.54 to 0.63, *p* < 0.001)	- Nurses emphasized the synergistic impact of transformational leadership, organizational justice, and quality of work life on their overall well-being and ability to provide high-quality patient care	- A holistic approach that addresses that leadership, justice, and work–life quality are needed to effectively promote nurse well-being and prevent burnout in healthcare settings

## Data Availability

The data will be available on request.
